# Identification of OmpA-Like Protein of *Tannerella forsythia* as an *O*-Linked Glycoprotein and Its Binding Capability to Lectins

**DOI:** 10.1371/journal.pone.0163974

**Published:** 2016-10-06

**Authors:** Toshi Horie, Megumi Inomata, Takeshi Into, Yoshiaki Hasegawa, Noriyuki Kitai, Fuminobu Yoshimura, Yukitaka Murakami

**Affiliations:** 1 Department of Oral Microbiology, Division of Oral Infections and Health Sciences, Asahi University School of Dentistry, Mizuho, Gifu, Japan; 2 Department of Orthodontics, Division of Oral Structure, Function and Development, Asahi University School of Dentistry, Mizuho, Gifu, Japan; 3 Department of Microbiology, School of Dentistry, Aichi Gakuin University, Nagoya, Aichi, Japan; University of Liverpool, UNITED KINGDOM

## Abstract

Bacterial glycoproteins are associated with physiological and pathogenic functions of bacteria. It remains unclear whether bacterial glycoproteins can bind to specific classes of lectins expressed on host cells. *Tannerella forsythia* is a gram-negative oral anaerobe that contributes to the development of periodontitis. In this study, we aimed to find lectin-binding glycoproteins in *T*. *forsythia*. We performed affinity chromatography of wheat germ agglutinin, which binds to *N*-acetylglucosamine (GlcNAc) and sialic acid (Sia), and identified OmpA-like protein as the glycoprotein that has the highest affinity. Mass spectrometry revealed that OmpA-like protein contains *O*-type *N*-acetylhexosamine and hexose. Fluorometry quantitatively showed that OmpA-like protein contains Sia. OmpA-like protein was found to bind to lectins including E-selectin, P-selectin, L-selectin, Siglec-5, Siglec-9, Siglec-10, and DC-SIGN. The binding of OmpA-like protein to these lectins, except for the Siglecs, depends on the presence of calcium. *N*-acetylneuraminic acid (NeuAc), which is the most abundant Sia, inhibited the binding of OmpA-like protein to all of these lectins, whereas GlcNAc and mannose only inhibited the binding to DC-SIGN. We further found that *T*. *forsythia* adhered to human oral epithelial cells, which express E-selectin and P-selectin, and that this adhesion was inhibited by addition of NeuAc. Moreover, adhesion of an OmpA-like protein-deficient *T*. *forsythia* strain to the cells was reduced compared to that of the wild-type strain. Our findings indicate that OmpA-like protein of *T*. *forsythia* contains *O*-linked sugar chains that can mediate interactions with specific lectins. This interaction is suggested to facilitate adhesion of *T*. *forsythia* to the surface of host cells.

## Introduction

Protein glycosylation generally occurs through attachment of glycan structures to proteins via asparagine residues (*N*-linked pathway) or serine/threonine residues (*O*-linked pathway). This post-translational modification has been recently found in bacterial species [1]. The gastrointestinal pathogen *Campylobacter jejuni* possesses an *N*-linked glycosylation pathway [2], whereas *Neisseria* species [3] and the gut symbiont *Bacteroides fragilis* [4] possesses *O*-linked glycosylation pathways. Protein glycosylation in bacteria is thought to occur at conserved glycosylation sequences: (D/E)X_1_NX_2_(S/T) (X_1_ and X_2_ can be any amino acids except for Pro) for *N*-linked glycosylation [2] and D(S/T)(A/I/L/V/M/T) for *O*-linked glycosylation [4, 5].

Bacterial glycoproteins are involved in many physiological functions of bacteria [1]. Most bacterial glycoproteins are surface-exposed, and surface appendages such as flagella and pili exist as glycoproteins. Several studies have shown that glycoproteins also play roles in aspects of pathogenicity, such as antigenic variation [6], stimulation of host immunity [7, 8], and resistance against cleavage by host proteases [9].

Previous studies have shown that bacterial glycoproteins are also involved in adhesion to host cells [10–13]. Furthermore, the glycoprotein Mfa1 of the periodontal pathogen *Porphyromonas gingivalis* has been reported to bind to the C-type lectin dendritic cell-specific intercellular adhesion molecule-3-grabbing non-integrin (DC-SIGN) expressed on the surface of dendritic cells, which mediates host-bacterium interactions [14, 15]. Lectins have the ability to recognize sugar chains and exist in a wide variety of organisms, including humans, microorganisms, and plants [16]. However, knowledge regarding the capability of bacterial glycoproteins to bind to lectins on the surface of host cells is still limited.

*Tannerella forsythia* is a gram-negative oral anaerobe that is recognized as a major contributor to periodontitis in humans [17, 18]. In this organism, S-layer and several proteins have been identified as glycoproteins [19, 20]. However, the functions of these glycoproteins, such as lectin binding, remain unclear. In this study, we aimed to investigate lectin-binding glycoproteins of *T*. *forsythia* by utilizing lectin affinity chromatography and mass spectrometry (MS). Here, we identify OmpA-like protein as a novel *O*-linked glycoprotein in *T*. *forsythia*. In addition, we show the characteristics of OmpA-like protein and its binding capability to lectins expressed on host cells.

## Materials and Methods

### Reagents and antibodies, and plasmids

Recombinant Fc-conjugated human IgG1, E-selectin, P-selectin, L-selectin, sialic acid-binding immunoglobulin-type lectin (Siglec)-3, Siglec-5, Siglec-7, Siglec-9, Siglec-10, Siglec-11, DC-SIGN, and CD161 were obtained from R&D Systems (Minneapolis, MN, USA). Anti-*O*-*N*-acetylglucosamine (GlcNAc) antibody (CTD110.6) was purchased from Cell Signaling Technology (Beverly, MA, USA). Anti-sialic acid (Sia) α2–3 monoclonal antibody (HYB4), GlcNAc, and *N*-acetylneuraminic acid (NeuAc) were obtained from Wako (Tokyo, Japan). Biotin-conjugated wheat germ agglutinin (WGA), *Maackia amurensis* (MAM), and WGA agarose were purchased from J-Oil Mills (Tokyo, Japan). Biotin-conjugated succinylated WGA was obtained from EY Laboratories (San Mateo, CA, USA). Fetuin and asialofetuin were purchased from Sigma-Aldrich (St. Louis, MO, USA). The plasmids of pCMF118 and RK231 were kindly provided by Prof. L. Comstock (Harvard Medical School, Boston, MA, USA) and Prof. M. Malamy (Tufts University, Boston, MA, USA), respectively.

### Bacterial strains and growth conditions

*T*. *forsythia* ATCC 43037 was used as a wild-type (WT) strain in this study. The mutant strain that lacks OmpA-like protein (Δ*1331*) was described previously [21]. These *T*. *forsythia* strains were grown anaerobically (10% CO_2_, 10% H_2_, and 80% N_2_) in trypticase soy broth (BD, Franklin Lakes, NJ, USA) containing 2.5 mg/ml yeast extract, 2.5 μg/ml hemin, 5 μg/ml menadione, 10 μg/ml *N*-acetylmuramic acid (Sigma-Aldrich), and 5% FBS (Nichirei Biosciences, Tokyo, Japan). *T*. *forsythia* was also grown on Brucella HK agar (Kyokuto Pharmaceutical Industrial, Tokyo, Japan), which was supplemented with 5% rabbit laked blood, 2.5 μg/ml hemin, 5 μg/ml menadione, and 10 μg/ml *N*-acetylmuramic acid (*Tf* agar). Bacterial growth was monitored by measuring OD_660_.

### Cell culture

Human oral epithelial Ho-1-n-1 cells, human umbilical vein endothelial cells (HUVECs), and HeLa cells were grown as described previously [22–24].

### Isolation of glycoproteins from *T*. *forsythi*a whole-cell lysates

To isolate lectin-binding glycoproteins in *T*. *forsythia* whole-cell lysates, we referred to a protocol that utilizes affinity chromatography of WGA, a representative plant lectin [25]. In brief, bacterial cells of *T*. *forsythia* were washed with PBS and then resuspended in 10 mM HEPES buffer (pH7.4) containing protease inhibitors (0.1 mM *N*α-*p*-tosyl-_L_-lysinechloromethylketone, 0.2 mM PMSF, and 0.1 mM leupeptin). The lysates were disrupted in a French pressure cell, and undisrupted bacterial cells were removed by centrifugation at 1,000 × *g* for 10 min. Whole-cell lysates were solubilized with 1% dodecyl maltoside (DM) and then centrifuged at 100,000 × *g* for 1 h. The supernatant was applied to a WGA affinity agarose column that had been equilibrated with WGA buffer (10 mM Tris-HCl (pH 7.4), 0.15 M NaCl, 0.03% DM, and protease inhibitors). The column was then washed with 5 column volumes of WGA buffer. Proteins adhering to the agarose were eluted by adding 5 column volumes of elution buffer (10 mM Tris-HCl (pH 7.4), 0.15 M NaCl, 0.03% DM, protease inhibitors, and 0.2 M GlcNAc). Isolated proteins were dialyzed and concentrated with Ficoll PM400 (GE Healthcare, Uppsala, Sweden) and a spin column (Thermo Fisher Scientific, Waltham, MA, USA). The isolated proteins were subjected to SDS-PAGE and were visualized with SyproRuby stain. Then, the purity of the isolated proteins was measured using ImageJ densitometry software (Version 1.6, National Institutes of Health, Bethesda, MD, USA).

### SDS-PAGE, protein staining, immunoblotting, and dot blotting

The samples were mixed with SDS sample buffer containing 2-mercaptoethanol, after which they were boiled and loaded onto a 10% gel. Proteins were visualized with Coomassie brilliant blue R-250 (CBB) stain or SyproRuby stain (Molecular Probes, Eugene, OR, USA). The glycoproteins were stained using the Pro-Q emerald 488 glycoprotein gel and blot stain kit (Molecular Probes). All Pro-Q emerald-stained gels were run with CandyCane glycoprotein molecular weight standards (Molecular Probes). For immunoblotting, proteins were transferred onto Immobilon-P transfer membranes (Millipore, Bedford, MA, USA). For dot blotting, samples were spotted onto nitrocellulose membranes (Advantec MFS, Pleasanton, CA, USA). These membranes were blocked, and immunoreactive bands and spots were detected using the antibodies, and visualized as described previously [25, 26]. Digital images were acquired using a Light-Capture II system (Atto, Tokyo, Japan) or a FluoroPhoreStar 3000 image capture system (Anatech, Tokyo, Japan).

### Protein analysis by MS

Isolated proteins were identified by matrix assisted laser desorption ionization-time of flight mass spectrometry (MALDI-TOF MS), as described previously [27]. Bands of interest were excised from SDS-PAGE gels. After in-gel tryptic digestion, the peptides were extracted and analyzed using a 4800 MALDI TOF/TOF analyzer (Applied Biosystems, Foster City, CA, USA). The identity of the proteins was deduced from the MS peaks by following peptide mass fingerprinting methods within Mascot (Matrix Science, Boston, MA, USA). Annotation and homology searches were performed using BLAST (http://blast.ncbi.nlm.nih.gov/Blast.cgi).

### Identification of *O*- and *N*-linked sugar chains in OmpA-like protein by liquid chromatography MS (LC-MS)

Glycosylation was determined by Sumitomo Bakelite (Tokyo, Japan). For analysis of *O*- and *N*-linked carbohydrates, sugar chains on glycoprotein bands excised from SDS-PAGE gels were released by hydrazinolysis. To analyze *O*-linked sugar chains, glycoproteins were digested with pronase before hydrazinolysis. Sugar chain purification and fluorescent labeling (2-aminobenzamide: 2AB) were performed with a BlotGlyco sugar chain purification kit (Sumitomo Bakelite). LC-MS was performed using an ultra-fast LCMS-IT-TOF (Shimadzu, Kyoto, Japan). Ten major peaks in the LC chromatogram were chosen, and the sugar chain composition was estimated using the GlycoMod tool (http://www.expasy.org/tools/glycomod/) and GlycoSuite database (http://glycosuitedb.expasy.org/glycosuite/glycodb). *O*-linked monosaccharides were analyzed in MS chromatograms, using representative monosaccharide m/z values.

### Quantitation of Sia in OmpA-like protein

Sia in OmpA-like protein was measured using a sialic acid quantitation kit (PROzyme, San Leandro, CA, USA) according to the manufacturer’s instructions. Fetuin and asialofetuin were used as the positive control and negative control, respectively. We also used sialidase-treated OmpA-like protein, which was treated with sialidase, followed by removal of Sia using a spin column. The fluorescence intensity (FI) of the samples and standards was measured using a Tecan Infinite M200 microplate reader (excitation, 530 nm; and emission, 590 nm; Durham, NC, USA). The results are representative of three independent experiments.

### Enzymatic deglycosylation of OmpA-like protein

To examine the type of glycosylation, OmpA-like protein was treated with *N*-glycanase (PROzyme), according to the manufacturer’s instructions. Fetuin was used as a positive control. Samples were then run on 10% SDS-PAGE gels and stained with SyproRuby and Pro-Q emerald 488.

### ELISA

The wells of 96-well microtiter plates were coated with 50 μl of 5 μg/ml OmpA-like protein in carbohydrate buffer (15 mM Na_2_CO_3_ and 35 mM NaHCO_3_ (pH 9.6)) for 18–24 h at 4°C. The wells were washed with PBS containing 0.1% Tween 20 (PBS-T) and blocked with 200 μl of blocking buffer (20 mM Tris-HCl (pH 7.4), 150 mM NaCl, and 1% BSA for 2 h at room temperature. After one wash with PBS-T, triplicate wells were incubated with each Fc-conjugated recombinant human lectin in 50 μl of buffer (10 mM Tris-HCl (pH 7.4), 150 mM NaCl, 10 mM CaCl_2_, 1% BSA, and 0.05% Tween 20) at concentrations of 10, 30, and 50 μg/ml for 3 h. After 5 washes with PBS-T, the wells were incubated with mouse monoclonal JDC-10 anti-human IgG1 Fc-HRP (Abcam, Cambridge, UK) for 1 h. After washing with PBS-T, the wells were incubated with 3,3',5,5'-tetramethylbenzidine liquid substrate at room temperature. The reaction was stopped by adding 0.5 N HCl, and color development was measured at 450 nm. For the carbohydrate blocking assay, 200 mM GlcNAc, 200 mM NeuAc, or 10 mM mannose was added to the wells for 2 h before addition of each Fc-conjugated recombinant human lectin at a concentration of 10 μg/ml for 3 h. To detect biotin-conjugated lectins, the wells of 96-well microtiter plates were coated with OmpA-like protein, sialidase-treated OmpA-like protein, fetuin or asialofetuin and blocked with blocking buffer. Then, the wells were incubated with 5 μg/ml control-biotin, WGA-biotin, succinylated WGA-biotin, or MAM-biotin for 3 h. After washing with PBS-T, the wells were incubated with high-sensitivity streptavidin-HRP (Thermo Fisher Scientific) for 1 h.

### RNA isolation and RT-PCR

Total RNA was prepared from cells using a GenElute mammalian total RNA miniprep kit (Sigma-Aldrich). Total RNA (1 μg) was reverse-transcribed using ReverTraAce reverse transcriptase (Toyobo, Otsu, Japan) with both an oligo21dT primer and random hexamer primers. The primers used for PCR reactions were as follows: human E-selectin: sense, 5ʹ-ATGCCTGTGTGAGCAAGCATTTA-3ʹ; and anti-sense, 5ʹ-AGGCTAGAGCAGCTTTGGCAATTA-3ʹ, human P-selectin: sense, 5ʹ-CCTTCAGGACAATGGACAGCAGTA-3ʹ; and anti-sense, 5ʹ-ATGCTGTTTGTGCAGAGCCATTA-3ʹ, and human β-actin: sense, 5ʹ-TGGCACCCAGCACAATGAA-3ʹ; and anti-sense, 5ʹ-CTAAGTCATAGTCCGCCTAGAAGCA-3ʹ.

These primer sets specifically detect the respective genes in PCR reactions. PCR was performed for 30 cycles, with each cycle consisting of 94°C for 30 sec, 55°C for 30 sec, and 68°C for 1 min. The PCR amplicons were visualized on 1.5% agarose gels stained with ethidium bromide, which were then photographed under UV light.

### Adherence assay

Ho-1-n-1 cells were washed and incubated in antibiotic-free medium containing 2% FBS for 6 h. *T*. *forsythia* WT or Δ*1331* was added at a m.o.i. of 100 and incubated for 3 h. Ho-1-n-1 cells were washed three times with sterile PBS and lysed by incubation in sterile water for 20 min. Cell lysates were diluted in sterile PBS and cultured on supplemented blood agar plates for 7 days. The number of bacteria was determined by counting colonies. For the carbohydrate blocking assay, cells were preincubated with 200 mM GlcNAc, 200 mM NeuAc, or 10 mM mannose for 2 h before WT or Δ*1331* infection. All assayswere performed in triplicate.

### Complementation of *tf1331* into *T*. *forsythia* Δ*1331*

The coding region of *tf1331* was amplified from DNA of *T*. *forsythia*, digested with BamHI, and ligated into the BamHI site of pCMF118, which is a pCMF6 [4] based plasmid harboring a strong promoter for the increased expression of cloned genes compared to pCMF6. The ribosome-binding site of *tf1331* was inserted before the start codon using a QuickChange II site-directed mutagenesis kit (Agilent Technologies, Palo Alto, CA, USA) according to the manufacturer’s instructions. The primers used for PCRs were as follows: *tf1331*: sense, 5ʹ-CGCGGATCCATGAAGACTAAGGTATTACTTTTAGCG-3ʹ; and anti-sense, 5ʹ- CGCGGATCCTTTATCGTCTGTATTCATGATAACC-3ʹ, and ribosome-binding site: sense, 5ʹ-CGAACGTTGGATCCTTAGGTTATTGTTTAATAAAATATGAATAGATTATGAAGACTAAGG-3ʹ; and anti-sense, 5ʹ- CCTTAGTCTTCATAATCTATTCATATTTTATTAAACAATAACCTAAGGATCCAACGTTCG-3ʹ. Underlining shows the BamHI site. The resulting plasmid was introduced into *E*. *coli* HST08 competent cells (Takara, Shiga, Japan). We confirmed that the DNA sequence of the insert did not result in any unintentional base changes after extraction of the plasmid from the transformants. The plasmid was transferred from *E*. *coli* HST08 to *T*. *forsythia* Δ*1331* by the conjugation method [4, 28] using *E*. *coli* RK231 [29], a broad-host-range mobilizing IncP plasmid. In brief, in a first conjugation, 300 μl of the cultures of *E*. *coli* HST08 containing the plasmid and *E*. *coli* RK231 were mixed, and the cells were centrifuged and plated on LB agar supplemented with ampicillin and kanamycin. Then, the positive clones were used in a second conjugation. Five milliliters of the culture of the positive clone (OD_660_ = 0.6) and 40 ml of the culture of *T*. *forsythia* Δ*1331* (OD_660_ = 0.3) were mixed, and the cells were centrifuged and spotted onto *Tf* agar. Following aerobic incubation for 4 h, the cells were collected and plated onto gentamycin/erythromycin-containing *Tf* agar, and incubated anaerobically for 10 days. Erythromycin-resistant transconjugants were verified by PCR and immunoblotting.

### Statistical analysis

Data are expressed as the mean ± standard deviation (SD) (n = 3). *P* values were calculated using Student’s *t* test and considered significant at *P* < 0.05 or 0.01.

## Results

### OmpA-like protein is a novel glycoprotein isolated by WGA affinity chromatography in *T*. *forsythia*

**Fig 1:**
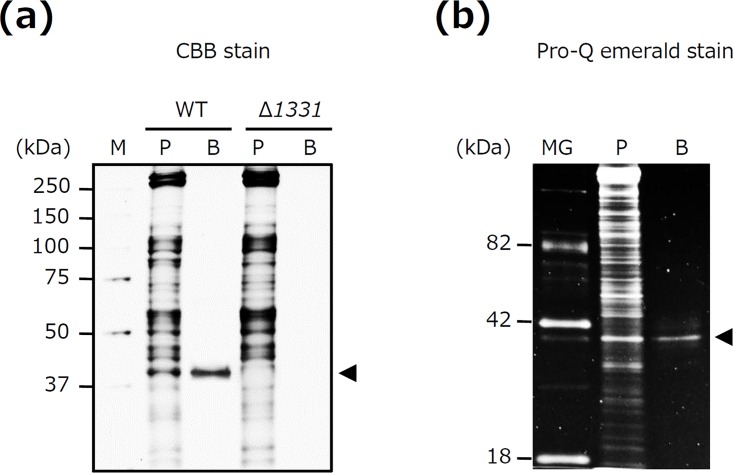
OmpA-like protein is a major glycoprotein isolated by WGA affinity chromatography. (a) Lectin-binding glycoproteins were isolated from wild-type (WT) *T*. *forsythia* and a *T*. *forsythia* deletion mutant that lacked OmpA-like protein (Δ*1331*) using WGA affinity chromatography. Isolated proteins were subjected to SDS-PAGE. The gels were stained with CBB. M, molecular marker; P, whole cell lysate prior to application to the WGA affinity column; B, proteins bound to the WGA column; arrowhead, OmpA-like protein. (b) Glycoproteins isolated from *T*. *forsythia* WT were subjected to SDS-PAGE. The gels were stained with Pro-Q emerald. MG, CandyCane glycoprotein molecular weight standards; P, whole-cell lysates prior to application to the WGA affinity column; B, proteins bound to the WGA column; arrowhead, OmpA-like protein.

We tried to isolate lectin-binding glycoproteins of *T*. *forsythia* by utilizing WGA affinity chromatography. WGA has the ability to bind to GlcNAc and Sia. Therefore, in the present study, the glycoproteins adhering to WGA were eluted using a buffer containing GlcNAc. Many bands were found for the *T*. *forsythia* whole-cell lysates prior to application to the WGA column, whereas the eluted sample only retained a single band with a molecular mass of 40 kDa (Fig 1A). No other bands were detected in the eluates (Fig 1A). This band was excised for analysis by MALDI-TOF MS, resulting in identification of OmpA-like protein (TF1331, BFO_0311, and Tanf_10935) (data not shown). The purity of the isolated OmpA-like protein was approximately 95% (S1 Fig). Of note, no such bands were detected in the eluates of Δ*1331* (*T*. *forsythia* mutant lacking OmpA-like protein) from the WGA column (Fig 1A). We next examined whether OmpA-like protein is indeed glycosylated. OmpA-like protein was visualized with Pro-Q emerald glycoprotein staining (Fig 1B). These results suggest that OmpA-like protein is a major glycoprotein isolated by WGA affinity chromatography in *T*. *forsythia*.

### OmpA-like protein has *O*-linked glycosylation

**Fig 2:**
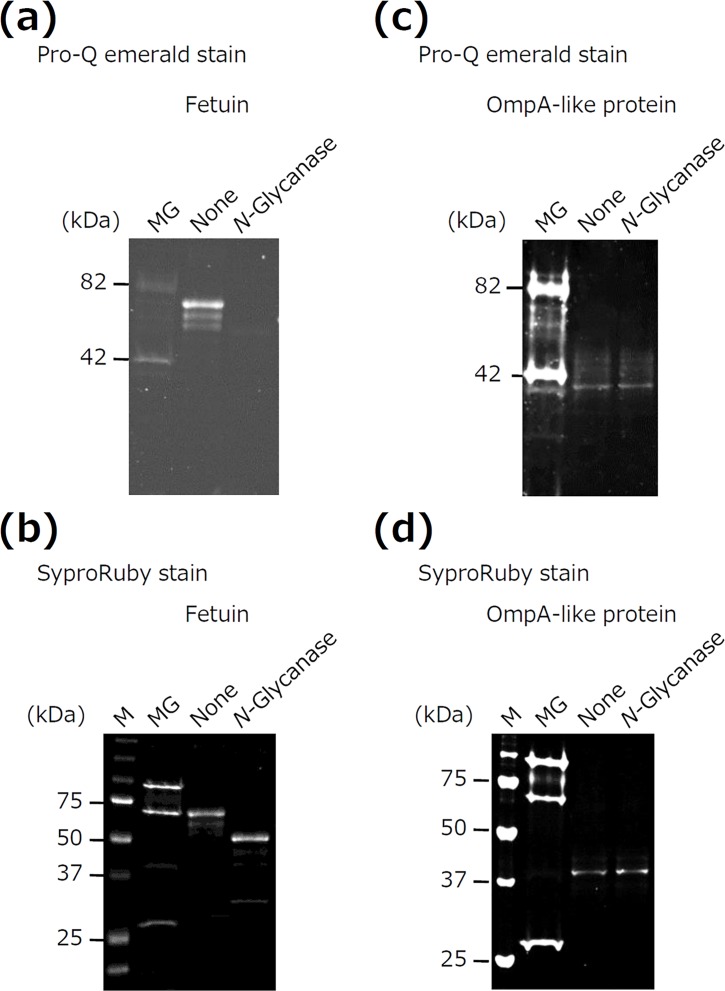
Enzymatic deglycosylation of OmpA-like protein. Fetuin (a, b) and OmpA-like protein (c, d) were treated with *N*-glycanase. The samples were then subjected to SDS-PAGE and stained with Pro-Q emerald and SyproRuby. M, molecular marker; MG; CandyCane glycoprotein molecular weight standards.

We examined the type of glycosylation occurring in OmpA-like protein by enzymatic deglycosylation with *N*-glycanase because *N*-glycanase treatment is known as the most effective method for removing *N*-linked sugar chains [30]. Fetuin was used as a positive control. *N*-glycanase treatment eliminated glycan attachment (Fig 2A) to fetuin and reduced the molecular mass (Fig 2B). In contrast, *N*-glycanase treatment did not alter the glycan attachment or molecular mass of OmpA-like protein (Fig 2C and 2D). These results suggest that OmpA-like protein may have *O*-linked sugar chains.

**Fig 3:**
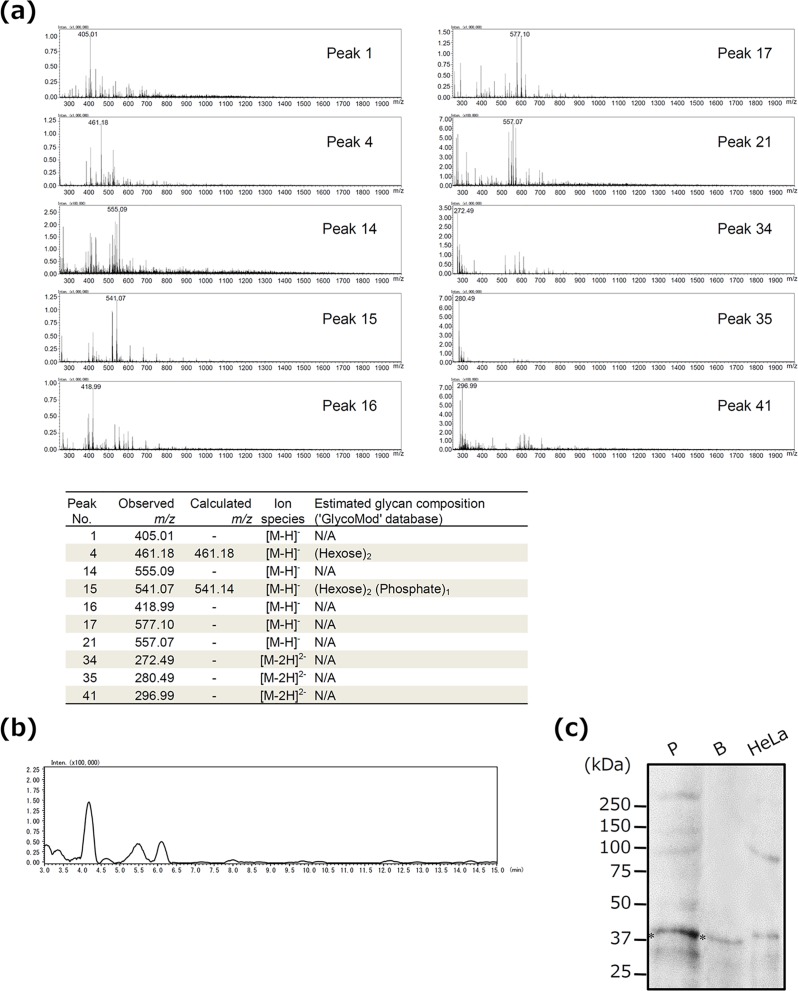
Identification of *O*- and *N*-linked sugar chains in OmpA-like protein by LC-MS. (a) Sugar chains released from OmpA-like protein were subjected to LC-MS to identify *O*-type sugar chains. Ten major peaks were chosen in the LC chromatogram, and the sugar chain composition was estimated using the GlycoMod tool and the GlycoSuite database. X-axis indicates m/z. Y-axis indicates signal intensity. No., number. N/A, not available. (b) Monosaccharides were analyzed by MS chromatograms using respective monosaccharide m/z values. This MS chromatogram was recorded by 340.1509, 2AB+HexNAc m/z value. X-axis indicates retention time. Y-axis indicates signal intensity. (c) *T*. *forsythia* whole-cell lysates before application to the column (P) and proteins bound to the WGA column (B) were analyzed by immunoblotting with an anti-*O*-GlcNAc antibody. HeLa cell lysate (HeLa) was used as a positive control. *, OmpA-like protein.

We next determined the type of sugar chains contained in OmpA-like protein by LC-MS for *N*-linked and *O*-linked glycosylation. *N*-linked sugar chains were not detected at all (data not shown). In contrast, analysis of 10 major peaks in the LC chromatogram for *O*-linked sugar chains revealed the presence of (hexose)_2_ in Peak No.4 and (hexose)_2_ (phosphate)_1_ in Peak No.15 (Fig 3A, S2 Fig). Determination of *O*-linked monosaccharides by MS chromatograms using representative monosaccharide m/z values also revealed that *N*-acetylhexosamine (HexNAc), which includes GlcNAc, was present during the early period of retention (Fig 3B). The presence of *O*-linked HexNAc in OmpA-like protein was also confirmed by its reactivity with *O*-GlcNAc antibody (Fig 3C). Thus, OmpA-like protein has *O*-linked glycans, including hexose, hexose phosphate, and HexNAc (especially GlcNAc).

**Fig 4:**
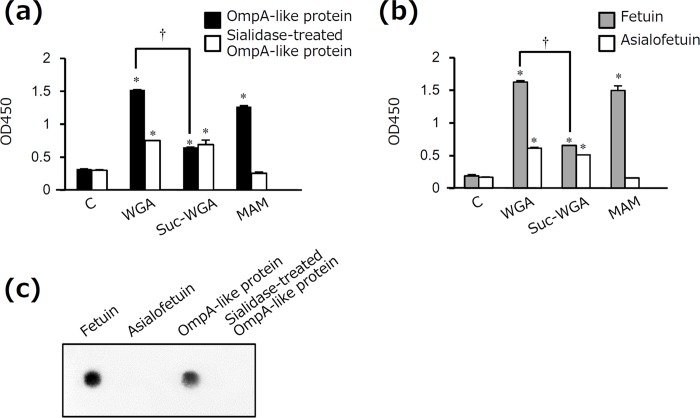
Detection of Sia in OmpA-like protein. (a, b) The wells of 96-well microtiter plates were coated with 5 μg/ml OmpA-like protein, sialidase-treated OmpA-like protein, fetuin, or asialofetuin for 18–24 h. After blocking, the wells were incubated with 5 μg/ml control (C)-biotin, WGA-biotin, succinylated WGA (Suc-WGA)-biotin, or MAM-biotin for 3 h. Binding was determined by ELISA using streptavidin-HRP. Data are expressed as the mean ± SD (n = 3). *, *P* < 0.01 for comparison with control-biotin. ^†^, *P* < 0.01 for comparison with WGA-biotin. (c) OmpA-like protein, sialidase-treated OmpA-like protein, fetuin, and asialofetuin were analyzed by dot blotting with anti-Siaα2–3 antibody.

OmpA-like protein was isolated by affinity chromatography of WGA, which recognizes GlcNAc and Sia; however, Sia was not detected in this protein by LC-MS. We therefore examined whether or not OmpA-like protein contains Sia by using several methods. In quantitative Sia analysis, Sia was detected in OmpA-like protein (6.5 μg/mg). Sia was also detected in fetuin (87.2 μg/mg, as described previously [31]). In contrast, Sia was not detected in sialidase-treated OmpA-like protein and asialofetuin (data not shown). In ELISA, OmpA-like protein bound to not only WGA but also succinylated WGA, which recognizes only GlcNAc (Fig 4A). However, the binding affinity of succinylated WGA to OmpA-like protein was considerably weaker than that of WGA (Fig 4A). In addition, MAM, which recognizes Siaα2-3-galactose (Gal), bound to OmpA-like protein similarly to WGA (Fig 4A). Sialidase-treated OmpA-like protein bound to WGA weakly, and it did not bind to MAM (Fig 4A). Fetuin and asialofetuin bound to WGA, succinylated WGA, and MAM, similarity to OmpA-like protein and sialidase-treated OmpA-like protein, respectively (Fig 4B). Furthermore, OmpA-like protein and fetuin, but not sialidase-treated protein and asialofetuin, reacted with the anti-Siaα2–3 antibody (Fig 4C). These results suggest that OmpA-like protein contains Sia residues. Because *N*-linked sugar chains were not detected at all in OmpA-like protein by LC-MS, *O*-linked Sia is present in this protein.

### OmpA-like protein binds to lectins expressed on surface of host cells

**Fig 5:**
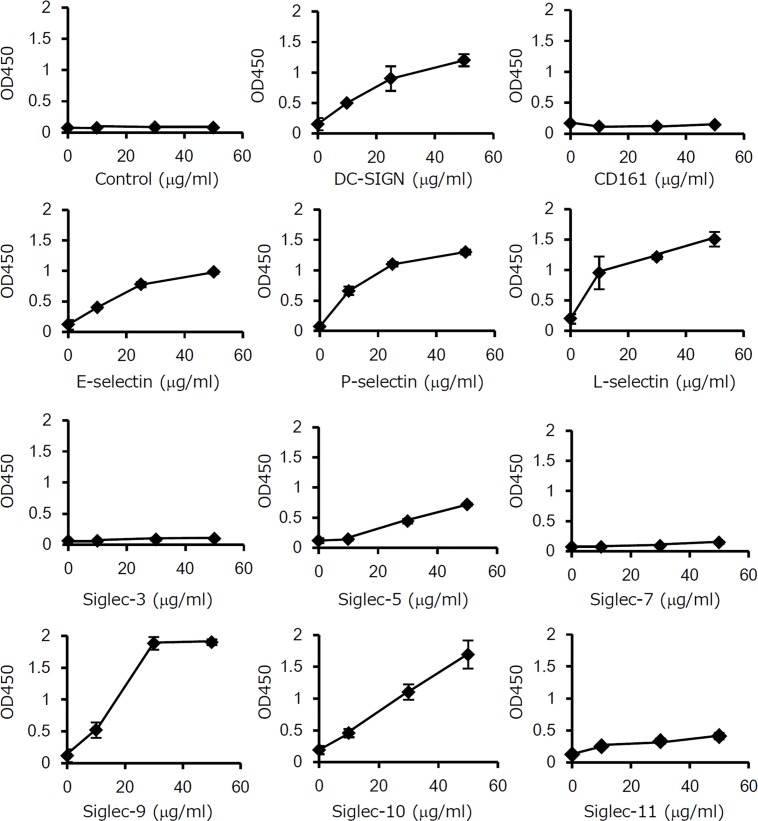
OmpA-like protein binds to lectins expressed on host cells. The wells of 96-well microtiter plates were coated with 5 μg/ml OmpA-like protein for 18–24 h. After blocking, the wells were incubated with Fc-conjugated recombinant protein (control IgG1, E-selectin, P-selectin, L-selectin, Siglec-3, Siglec-5, Siglec-7, Siglec-9, Siglec-10, Siglec-11, DC-SIGN, or CD161) at 10, 30, and 50 μg/ml for 3 h. IgG1/Fc was used as a negative control. Binding was determined by ELISA using anti-human IgG Fc secondary antibody conjugated with HRP. The results are expressed as the mean ± SD (n = 3).

The bacterial glycoprotein Mfa1 of *P*. *gingivalis* has been reported to bind to DC-SIGN expressed on the surface of dendritic cells, mediating host-bacterium interactions [14, 15]. We therefore examined whether or not OmpA-like protein binds to cell surface lectins of human cells. The ELISA-based in vitro analysis revealed that all types of selectins (E-selectin, P-selectin, and L-selectin) bound to OmpA-like protein (Fig 5). Among Siglecs, Siglec-5, -9, and -10, but not Siglec-3 and -7, bound to OmpA-like protein. Siglec-11 weakly bound to OmpA-like protein. DC-SIGN was also found to bind to OmpA-like protein, but CD161 did not. These results indicate that OmpA-like protein has the capability to bind to specific lectins.

**Fig 6:**
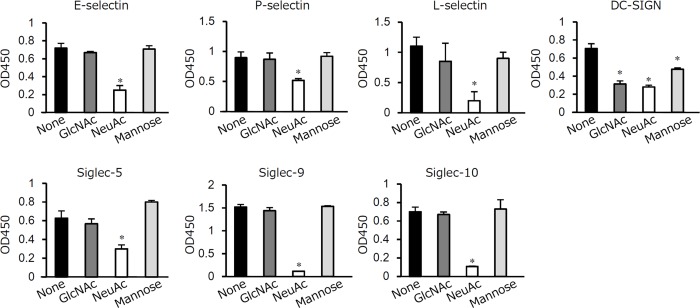
Sugar chains of OmpA-like protein contribute to lectin binding. The wells of 96-well microtiter plates were coated with 5 μg/ml OmpA-like protein for 18–24 h. After blocking, 200 mM GlcNAc, 200 mM NeuAc, or 10 mM mannose was added to the wells for 2 h before addition of 10 μg/ml Fc-conjugated recombinant proteins for 3 h. Binding was determined by an Fc-specific ELISA. The results are expressed as the mean ± SD (n = 3). *, *P* < 0.01.

E-selectin, P-selectin, and L-selectin are C-type lectins that belong to the selectin family and recognize sialylated carbohydrates [32]. Siglecs are I-type lectins that recognize glycans containing Sia [33]. DC-SIGN is a C-type lectin that has high affinity for mannose-containing carbohydrate [34]. DC-SIGN also recognizes GlcNAc [35] and Sia residues [36]. To determine whether these lectins recognize the sugar chains of OmpA-like protein, a carbohydrate inhibition assay was performed. Of note, NeuAc, the most common Sia [37], competed with OmpA-like protein for binding with all lectins, whereas GlcNAc and mannose only competed for binding to DC-SIGN (Fig 6). C-type lectins (E-selectin, P-selectin, L-selectin, and DC-SIGN) have been reported to bind to carbohydrates depending on the presence of calcium [32, 38]. We examined whether or not interactions of these C-type lectins with OmpA-like protein are dependent on calcium. EGTA, a calcium chelator, inhibited the binding of OmpA-like protein to these lectins (S3 Fig). In contrast, EGTA did not affect the binding to Siglecs (S3 Fig).

### Sugar chains of OmpA-like protein mediate adhesion of *T*. *forsythia* to human oral epithelial cells

**Fig 7:**
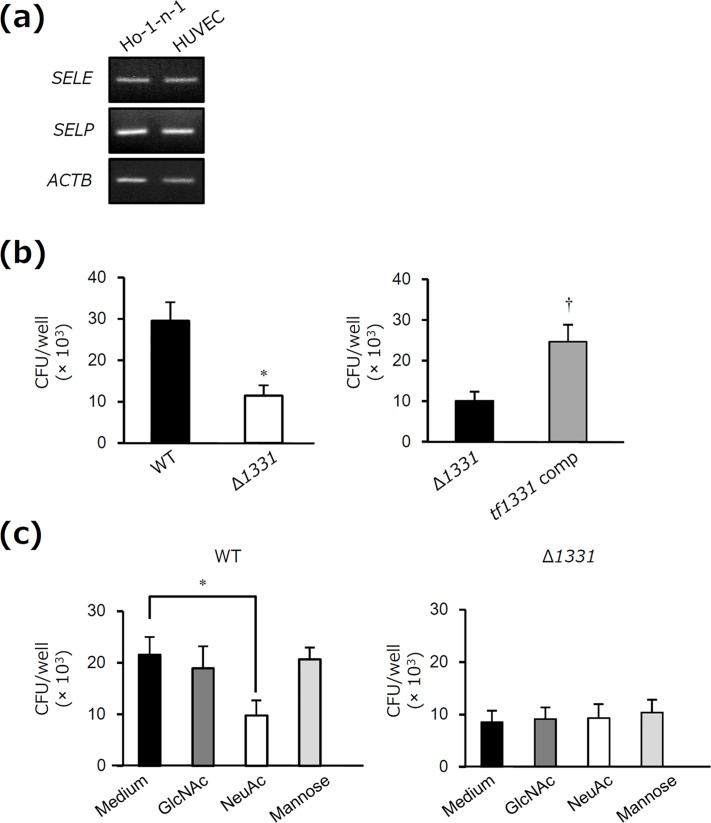
Sugar chains of OmpA-like protein mediate adhesion of *T*. *forsythia* to human oral epithelial cells. (a) Total RNA (1 μg) was prepared from Ho-1-n-1 cells and HUVECs to assess the expression of *SELE* (E-selectin), *SELP* (P-selectin), and *ACTB* (β-actin) by RT-PCR. HUVECs, which express E-selectin and P-selectin mRNA, were used as positive controls. The PCR products were visualized on 1.5% agarose gels stained with ethidium bromide and photographed under UV light. The results are representative of three independent experiments. (b) WT, Δ*1331* or Δ*1331* complemented with *tf1331* (*tf1331* comp) was added to Ho-1-n-1 cells at a m.o.i. of 100 for 3 h. At the end of the incubation period, the cells were washed three times with PBS and lysed by incubation in sterile water for 20 min. Cell lysates were serially diluted in sterile PBS and cultured on supplemented blood agar plates. The number of viable adherent bacteria was determined by counting the colonies that appeared. The results are expressed as the mean ± SD (n = 3). *, *P* < 0.05 for comparison with WT. ^†^, *P* < 0.05 for comparison with Δ*1331*. (c) Ho-1-n-1 cells were preincubated with and without 200 mM GlcNAc, 200 mM NeuAc, and 10 mM mannose for 2 h. Then, WT or Δ*1331* was added at a m.o.i. of 100 for 3 h, and the number of viable adherent bacteria was determined. The results are expressed as the mean ± SD (n = 3). *, *P* < 0.05.

Interactions between *T*. *forsythia* and oral epithelial cells are essential aspects of periodontal infections [39, 40]. We examined whether or not OmpA-like protein mediates adhesion of *T*. *forsythia* to oral epithelial cells. Human oral epithelial Ho-1-n-1 cells expressed both E-selectin and P-selectin mRNA, similarly to HUVECs (Fig 7A). We found that the WT strain of *T*. *forsythia* adhered to the cells (Fig 7B). In contrast, adhesion of the Δ*1331* mutant strain to the cells was reduced compared to that of the WT strain (Fig 7B). The complemented strain was constructed in which Δ*1331* was complemented with *tf1331* (S4 Fig), and adhesion of this strain was restored compared to that of the Δ*1331* mutant strain (Fig 7B). Moreover, adhesion of the WT strain to the cells was inhibited by addition of NeuAc, but not by addition of GlcNAc and mannose (Fig 7C). In contrast, adhesion of the Δ*1331* mutant strain to the cells was not inhibited by addition of NeuAc (Fig 7C). These results suggest that sugar chains of OmpA-like protein are involved in interactions of *T*. *forsythia* with oral epithelial cells.

## Discussion

In the present study, we provide the first evidence that OmpA-like protein is a novel *O*-linked glycoprotein isolated by WGA affinity chromatography in *T*. *forsythia*. We further found that OmpA-like protein specifically binds to lectins expressed on the surface of host cells, including E-selectin, P-selectin, L-selectin, Siglec-5, Siglec-9, Siglec-10, and DC-SIGN. *T*. *forsythia* adhered to oral epithelial cells that express E-selectin and P-selectin, and this adhesion was inhibited by NeuAc. Moreover, adhesion of OmpA-like protein-deleted *T*. *forsythia* to the cells was reduced compared to that of the WT organism. Therefore, our findings suggest that interaction of *O*-linked glycosylated OmpA-like protein with lectins partly mediates adhesion of *T*. *forsythia* to host cells.

In this study, we characterized the sugar chains of *T*. *forsythia* OmpA-like protein. First, we estimated that OmpA-like protein contained S/T-*O*-GlcNAc, given that LC-MS indicated the presence of *O*-type HexNAc and the absence of any *N*-type sugar chains (Fig 3A and 3B and data not shown), and also based on its reactivity with an *O*-GlcNAc antibody that recognizes S/T-*O*-GlcNAc (Fig 3C). Second, OmpA-like protein contained Sia residues, based on the results of quantitative Sia analysis. In particular, we estimated that Siaα2-3-Galβ1-4GlcNAc was present on the terminal of the sugar chains based on its reactivity with an anti-Siaα2–3 antibody, which recognizes Siaα2-3-Galβ1-4GlcNAc presented on the terminal of sugar chains (Fig 4C); MAM, which recognizes Siaα2-3-Gal (Fig 4A); Siglec-5, -9, and -10 (Fig 5); and selectins (Fig 5). Siglec-5, -9, and -10 strongly bind to Siaα2-3-Galβ1-4GlcNAc, which is known to only weakly bind to Siglec-3, -7, and -11 [33]. Siglec-3 is known to strongly interact with Siaα2-6-Galβ1-4GlcNAc, and Siglec-7 and -11 interact with Siaα2-8-Siaα2-3-Galβ1-4GlcNAc [33]. Selectins are known to recognize Sia when Siaα2-3-Gal is present in glycan [41]. Third, we estimated that OmpA-like protein contained hexose, especially mannose, and hexose phosphate based on the LC-MS results (Fig 3A) and its reactivity with DC-SIGN (Fig 5). We speculated that several types of sugar chain may be present on OmpA-like protein.

We detected Sia in OmpA-like protein by ELISA and dot blotting (Fig 4A and 4C); however, Sia was not detected in OmpA-like protein by LC-MS (Fig 3). This lack of detection may be attributed to the effects of hydrazinolysis. Patel et al. reported that hydrazinolysis causes Sia to undergo *N*-deacetylation, which results in a loss of information regarding sialylated oligosaccharides, although hydrazinolysis is the chemical and enzymatic method best capable of releasing all (*N*-linked and *O*-linked) oligosaccharides from a glycoprotein [42]. This lack of detection may also be at least partly attributed to Sia being a high-molecular-mass monosaccharide and to the existence of approximately 40 derivatives in the Sia family [43].

*O*-linked HexNAc was detected in the MS chromatograms during the early period of retention (from 4 to 4.5 min) (Fig 3B), but it was not detected in the LC chromatogram (Fig 3A). This is probably because LC chromatography does not typically detect glycans during such an early period of retention. Indeed, we obtained many peaks after 10 min of retention time in the LC chromatogram (S2 Fig).

*T*. *forsythia* belongs to the order *Bacteroidales* within the *Bacteroidetes* phylum of bacteria. The conserved D(S/T)(A/I/L/V/M/T) motif for *O*-linked glycosylation has been identified in *Bacteroidetes* species [4, 5]. This motif was found in OmpA-like protein of *T*. *forsythia*. We also found that this protein contains 24 serine and nine threonine residues, which can function as *O*-linked glycosylation sites. Future studies should determine the glycosylation sites for *T*. *forsythia* OmpA-like protein.

In the present study, we showed that adhesion of OmpA-like protein-deficient *T*. *forsythia* to oral epithelial cells was reduced compared to that of the WT organism (Fig 7B). Furthermore, adhesion of the WT strain, but not OmpA-like protein-deficient *T*. *forsythia*, to the cells was inhibited by NeuAc (Fig 7C). These results suggest that adhesion of *T*. *forsythia* to oral epithelial cells is partly dependent on the interaction of sugar chains of OmpA-like protein with lectins, although it is also necessary to consider other characteristics of OmpA-like protein-deleted *T*. *forsythia*, such as cell swelling and cell structural changes [21]. In our previous study, we showed that adhesion of S-layer-deficient *T*. *forsythia* to oral epithelial cells was considerably decreased compared to that of the WT strain [44]. These observations suggest that S-layer also plays an important role in adhesion.

In the innate immune response, the lectin-mediated host-bacteria interaction initiates the immune response to eliminate bacteria [16, 45]. In contrast, a recent study suggested that interaction of glycan epitopes on bacteria with lectins mediates bacterial persistence in the host. The glycoprotein Mfa1 of *P*. *gingivalis* binds to DC-SIGN, which results in inhibition of cytokine secretion from dendritic cells [15]. Because *T*. *forsythia* is present in periodontal pockets, this organism may modulate the host immune response through binding to lectins expressed on host cells, leading to the development of periodontitis.

In a previous study, we isolated a glycoprotein in *P*. *gingivalis*, leading to the identification of OmpA-like protein [25]. However, it remains unclear whether bacterial glycoproteins, including *P*. *gingivalis* OmpA-like protein, can bind to specific classes of lectins expressed on host cells. Furthermore, the identity of the lectin-binding glycoproteins of *T*. *forsythia* is also unclear. In conclusion, we provide the first evidence that the *O*-glycosylated OmpA-like protein of *T*. *forsythia* binds to specific lectins expressed on host cells, and may mediate adhesion to lectin-expressing oral epithelial cells. Further work is required to determine whether this interaction regulates the host immune response to facilitate *T*. *forsythia* persistence.

## Supporting Information

S1 Fig Purification of isolated OmpA-like protein. *T*. *forsythia* whole-cell lysates prior to application to the WGA affinity column and 12 μg of isolated proteins (a), 16 μg and 8 μg of isolated proteins (b) were subjected to SDS-PAGE and visualized with SyproRuby stain. Then, the purity of isolated proteins was measured using ImageJ densitometry software. The numbers shown above the peaks indicate the proportion (%). M, molecular marker.
(PPTX)

S2 Fig LC chromatogram of OmpA-like protein by LC-MS. OmpA-like protein isolated by WGA column chromatography was subjected to LC-MS to identify *O*-type sugar chains. An LC chromatogram of OmpA-like protein was obtained by LC-MS. X-axis indicates retention time. Y-axis indicates fluorescence intensity. The numbers in the LC chromatogram indicate the peak number.
(PPTX)

S3 Fig Calcium ion-dependent interaction of OmpA-like protein with C-type lectins. The wells of 96-well microtiter plates were coated with 5 μg/ml OmpA-like protein for 18–24 h. After blocking, the wells were preincubated with EGTA or 1 N NaOH for 1 h. Then, the wells were incubated with 10 μg/ml Fc-conjugated recombinant proteins for 3 h. After washing, binding was determined by Fc-specific ELISA. The results are expressed as the mean ± SD (n = 3). *, *P* < 0.01.
(PPTX)

S4 Fig Expression of OmpA-like protein in Δ*1331* complemented with *tf1331*. (a) Verification of the gene complementation by PCR using *tf1331* primers. The PCR amplicons were visualized on 1% agarose gels stained with ethidium bromide, which were then photographed under UV light. M, marker. 1, *T*. *forsythia* WT whole-cell lysates; 2, *T*. *forsythia* Δ*1331* whole-cell lysates; 3, Δ*1331* complemented with *tf1331* whole-cell lysates. (b) Verification of the gene complementation by immunoblotting with an anti-OmpA-like protein serum. 1, *T*. *forsythia* WT whole-cell lysates; 2, *T*. *forsythia* Δ*1331* whole-cell lysates; 3, Δ*1331* complemented with *tf1331* whole-cell lysates.
(PPTX)
