# Supplementary material for: Identification of OmpA-Like Protein of Tannerella forsythia as an O-Linked Glycoprotein and Its Binding Capability to Lectins
Source: PLoS One. 2016 Oct 6;11(10):e0163974. doi: 10.1371/journal.pone.0163974 (PMC5053532; doi:10.1371/journal.pone.0163974)
Supplement: S1 Fig — T. forsythia whole-cell lysates prior to application to the WGA affinity column and 12 μg of isolated proteins (a), 16 μg and 8 μg of isolated proteins (b) were subjected to SDS-PAGE and visualized with SyproRuby stain. Then, the purity of isolated proteins was measured using ImageJ densitometry software. The numbers shown above the peaks indicate the proportion (%). M, molecular marker. (PPTX) [file pone.0163974.s001.pptx]

## Slide 1
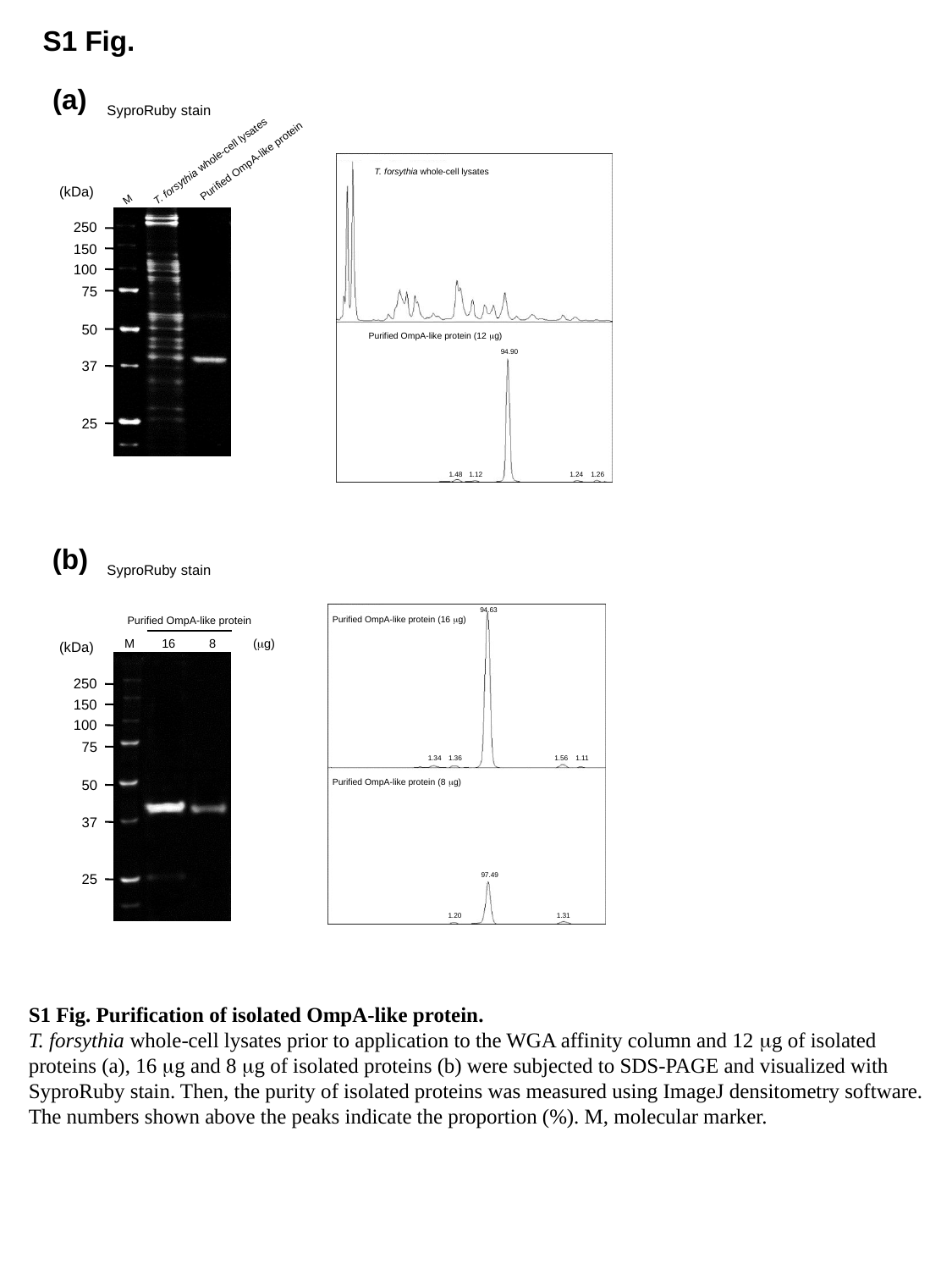

S1 Fig.
(a)
SyproRuby stain
T. forsythia whole-cell lysates
Purified OmpA-like protein
T. forsythia whole-cell lysates
(kDa)
M
250
150
100
75
50
Purified OmpA-like protein (12 mg)
94.90
37
25
1.48
1.12
1.24
1.26
(b)
SyproRuby stain
94.63
Purified OmpA-like protein (16 mg)
Purified OmpA-like protein
M
16
8
(mg)
(kDa)
250
150
100
75
1.34
1.36
1.56
1.11
50
Purified OmpA-like protein (8 mg)
37
97.49
25
1.20
1.31
S1 Fig. Purification of isolated OmpA-like protein.
T. forsythia whole-cell lysates prior to application to the WGA affinity column and 12 mg of isolated proteins (a), 16 mg and 8 mg of isolated proteins (b) were subjected to SDS-PAGE and visualized with SyproRuby stain. Then, the purity of isolated proteins was measured using ImageJ densitometry software. The numbers shown above the peaks indicate the proportion (%). M, molecular marker.
