# Supplementary material for: Identification of OmpA-Like Protein of Tannerella forsythia as an O-Linked Glycoprotein and Its Binding Capability to Lectins
Source: PLoS One. 2016 Oct 6;11(10):e0163974. doi: 10.1371/journal.pone.0163974 (PMC5053532; doi:10.1371/journal.pone.0163974)
Supplement: S2 Fig — OmpA-like protein isolated by WGA column chromatography was subjected to LC-MS to identify O-type sugar chains. An LC chromatogram of OmpA-like protein was obtained by LC-MS. X-axis indicates retention time. Y-axis indicates fluorescence intensity. The numbers in the LC chromatogram indicate the peak number. (PPTX) [file pone.0163974.s002.pptx]

## Slide 1
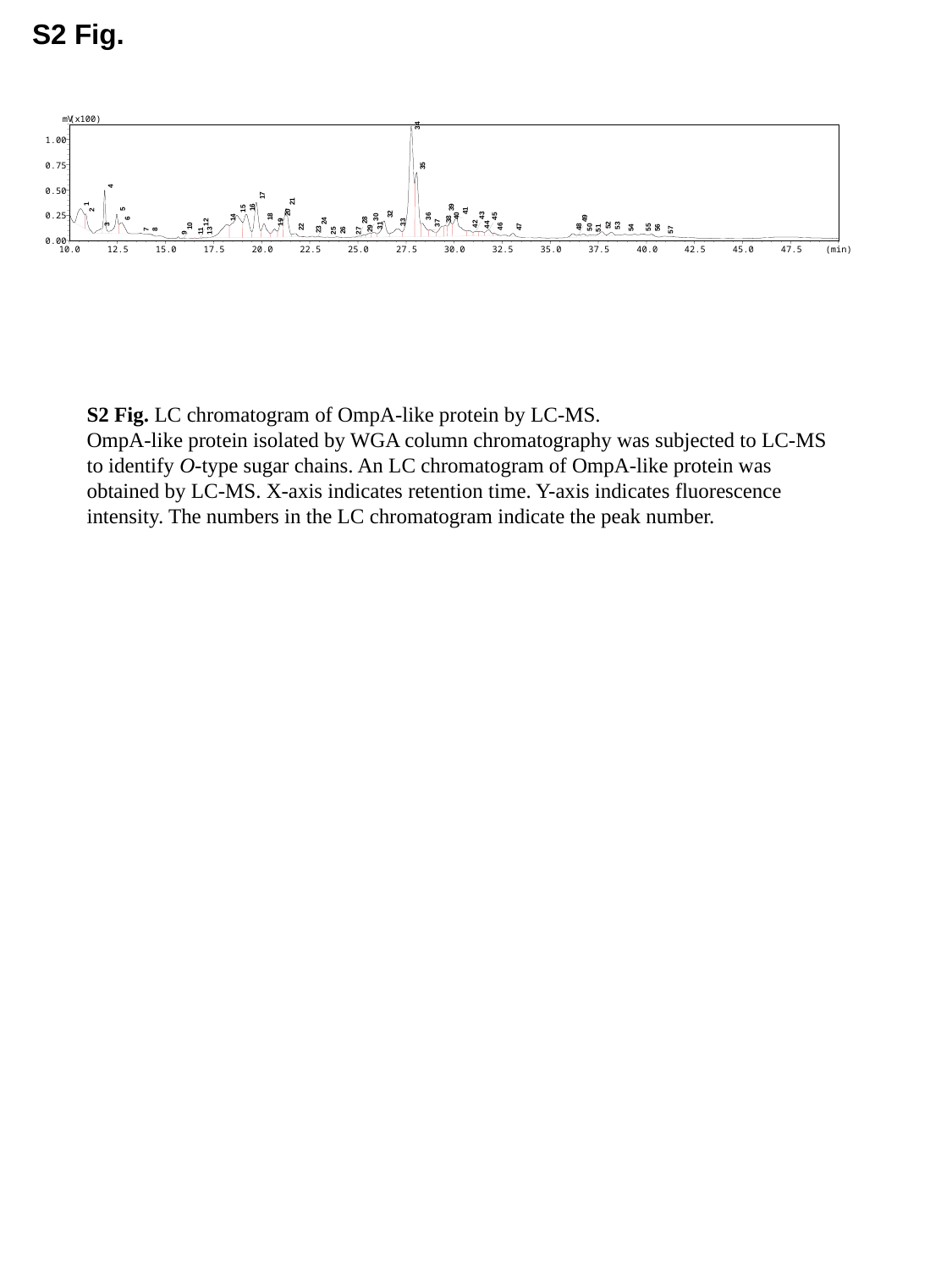

S2 Fig.
mV
(x100)
34
1.00
0.75
35
4
0.50
17
21
1
16
39
15
5
2
41
20
32
0.25
43
40
45
36
18
30
14
6
49
38
28
24
33
12
19
37
42
3
44
52
31
53
10
46
47
22
48
50
55
54
56
51
29
7
23
8
57
26
25
27
13
11
9
0.00
10.0
12.5
15.0
17.5
20.0
22.5
25.0
27.5
30.0
32.5
35.0
37.5
40.0
42.5
45.0
47.5
(min)
S2 Fig. LC chromatogram of OmpA-like protein by LC-MS.
OmpA-like protein isolated by WGA column chromatography was subjected to LC-MS to identify O-type sugar chains. An LC chromatogram of OmpA-like protein was obtained by LC-MS. X-axis indicates retention time. Y-axis indicates fluorescence intensity. The numbers in the LC chromatogram indicate the peak number.
