# Supplementary material for: Identification of OmpA-Like Protein of Tannerella forsythia as an O-Linked Glycoprotein and Its Binding Capability to Lectins
Source: PLoS One. 2016 Oct 6;11(10):e0163974. doi: 10.1371/journal.pone.0163974 (PMC5053532; doi:10.1371/journal.pone.0163974)
Supplement: S3 Fig — The wells of 96-well microtiter plates were coated with 5 μg/ml OmpA-like protein for 18–24 h. After blocking, the wells were preincubated with EGTA or 1 N NaOH for 1 h. Then, the wells were incubated with 10 μg/ml Fc-conjugated recombinant proteins for 3 h. After washing, binding was determined by Fc-specific ELISA. The results are expressed as the mean ± SD (n = 3). *, P < 0.01. (PPTX) [file pone.0163974.s003.pptx]

## Slide 1
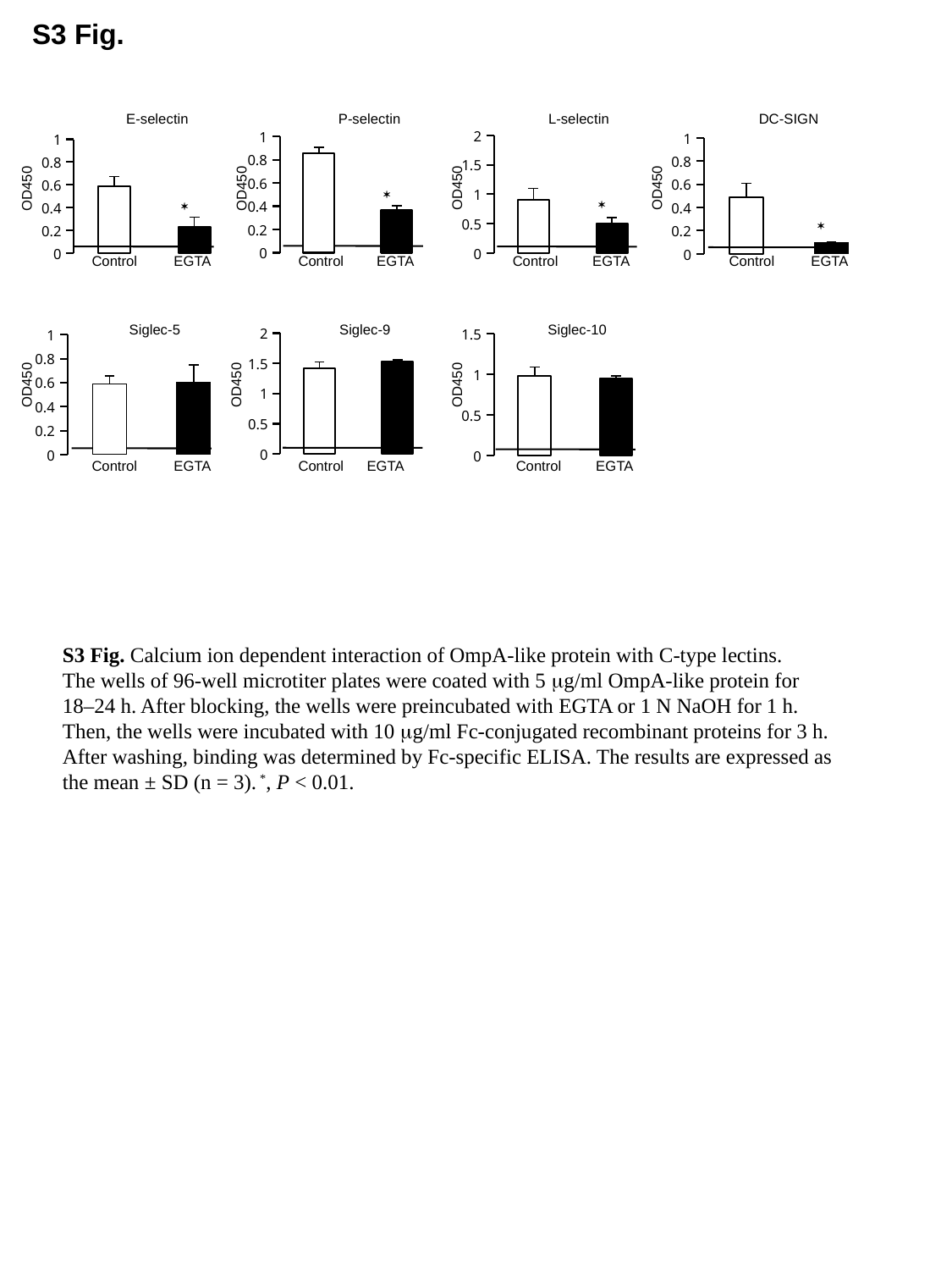

S3 Fig.
E-selectin
P-selectin
L-selectin
DC-SIGN
### Chart
| Category | |
|---|---|
### Chart
| Category | |
|---|---|
### Chart
| Category | |
|---|---|
### Chart
| Category | |
|---|---|OD450
OD450
OD450
OD450
*
*
*
*
Control
EGTA
Control
EGTA
Control
EGTA
Control
EGTA
Siglec-5
Siglec-9
Siglec-10
### Chart
| Category | |
|---|---|
### Chart
| Category | |
|---|---|
### Chart
| Category | |
|---|---|OD450
OD450
OD450
Control
EGTA
Control
 EGTA
Control
EGTA
S3 Fig. Calcium ion dependent interaction of OmpA-like protein with C-type lectins.
The wells of 96-well microtiter plates were coated with 5 mg/ml OmpA-like protein for 18–24 h. After blocking, the wells were preincubated with EGTA or 1 N NaOH for 1 h. Then, the wells were incubated with 10 mg/ml Fc-conjugated recombinant proteins for 3 h. After washing, binding was determined by Fc-specific ELISA. The results are expressed as the mean ± SD (n = 3). *, P < 0.01.
