# Supplementary material for: Identification of OmpA-Like Protein of Tannerella forsythia as an O-Linked Glycoprotein and Its Binding Capability to Lectins
Source: PLoS One. 2016 Oct 6;11(10):e0163974. doi: 10.1371/journal.pone.0163974 (PMC5053532; doi:10.1371/journal.pone.0163974)
Supplement: S4 Fig — (a) Verification of the gene complementation by PCR using tf1331 primers. The PCR amplicons were visualized on 1% agarose gels stained with ethidium bromide, which were then photographed under UV light. M, marker. 1, T. forsythia WT whole-cell lysates; 2, T. forsythia Δ1331 whole-cell lysates; 3, Δ1331 complemented with tf1331 whole-cell lysates. (b) Verification of the gene complementation by immunoblotting with an anti-OmpA-like protein serum. 1, T. forsythia WT whole-cell lysates; 2, T. forsythia Δ1331 whole-cell lysates; 3, Δ1331 complemented with tf1331 whole-cell lysates. (PPTX) [file pone.0163974.s004.pptx]

## Slide 1
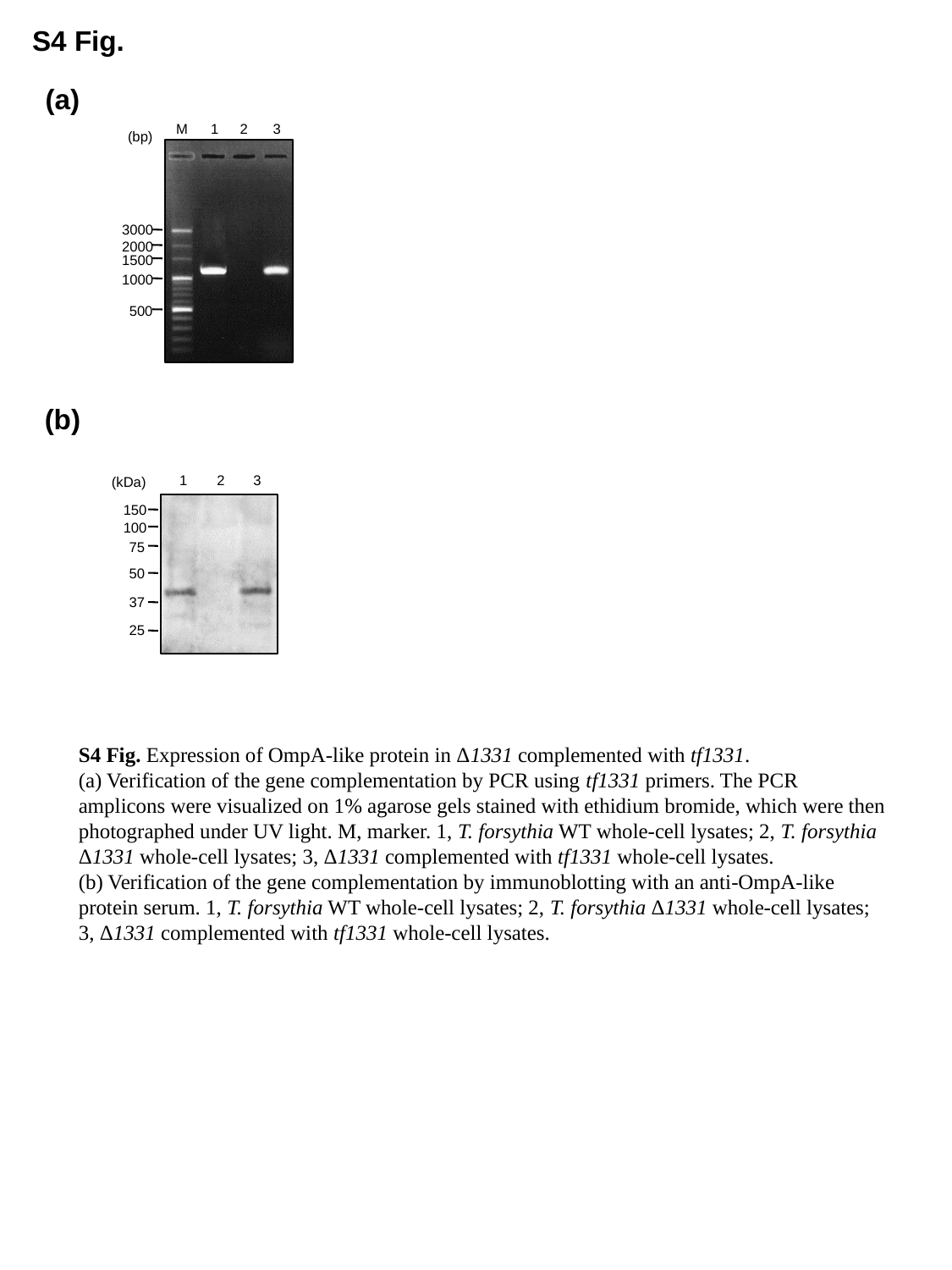

S4 Fig.
(a)
M
1
2
3
(bp)
3000
2000
1500
1000
500
(b)
1
2
3
(kDa)
150
100
75
50
37
25
S4 Fig. Expression of OmpA-like protein in Δ1331 complemented with tf1331.
(a) Verification of the gene complementation by PCR using tf1331 primers. The PCR amplicons were visualized on 1% agarose gels stained with ethidium bromide, which were then photographed under UV light. M, marker. 1, T. forsythia WT whole-cell lysates; 2, T. forsythia Δ1331 whole-cell lysates; 3, Δ1331 complemented with tf1331 whole-cell lysates.
(b) Verification of the gene complementation by immunoblotting with an anti-OmpA-like protein serum. 1, T. forsythia WT whole-cell lysates; 2, T. forsythia Δ1331 whole-cell lysates; 3, Δ1331 complemented with tf1331 whole-cell lysates.
